# Glucose-6-phosphate dehydrogenase neutralizes stresses by supporting reductive glutamine metabolism and AMPK activation

**DOI:** 10.1038/s41392-020-00399-x

**Published:** 2021-02-04

**Authors:** Benfu Zhong, Dewei Jiang, Yang Hong, Lifang Li, Li Qiu, Ronghui Yang, Xiaohan Jin, Yawen Song, Ceshi Chen, Binghui Li

**Affiliations:** 1 Department of Cancer Cell Biology and National Clinical Research Center for Cancer, Tianjin Medical University Cancer Institute and Hospital, Tianjin, 300060 P. R. China; 2 Department of Biochemistry and Molecular Biology, Capital Medical University, Beijing, 100069 P.R. China; 3 Key Laboratory of Animal Models and Human Disease Mechanisms of Chinese Academy of Sciences and Yunnan Province, Kunming Institute of Zoology, Chinese Academy of Sciences, Kunming, 650223 China

**Keywords:** Cell biology, Biochemistry

**Dear Editor**,

Glucose-6-phosphate dehydrogenase (G6PD) is the rate-limiting enzyme in the oxidative pentose phosphate pathway (oxPPP) that can generate cytosolic NADPH (Fig. 1a) for biosynthesis and oxidative defence. Here, we reveal a previously unidentified function of G6PD. It, even the natural G6PD deficiency-associated mutant without the activity to maintain the normal oxPPP, can antagonize the stresses by supporting the reductive glutamine metabolism and AMPK activation, independently of the NADPH generation by the oxPPP.

**Fig. 1:**
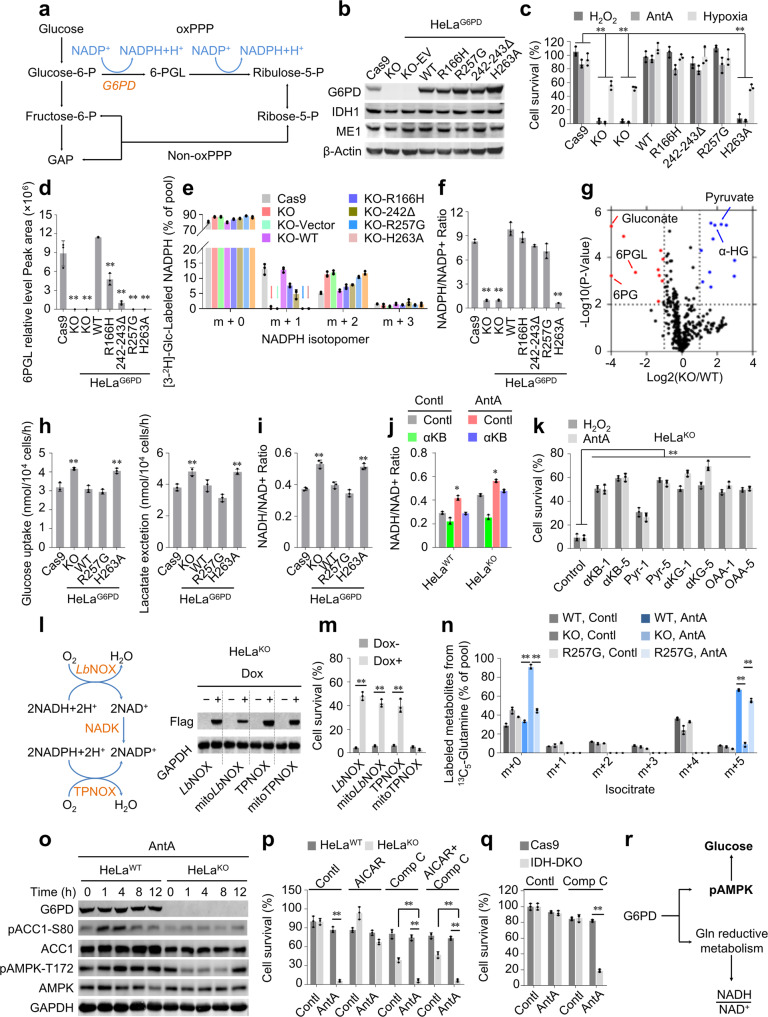
G6PD neutralizes stresses by supporting reductive glutamine metabolism and AMPK activation. **a** A simple schematic for the oxidative PPP and the non-oxidative PPP. G6PD enzymatic activity-deficiency alleles are well tolerated, except that it could bring a risk of acute non-spherocytic hemolytic anemia triggered by exogenous oxidative stressors in red blood cells. Based on the report from the World Health Organization, G6PD deficiency-associated variants are divided into several types, class-I G6PD mutants with an activity often less than 1% of normal are associated with chronic NSHA; class-II have an activity less than 10% of normal; class-III show 10-60% of residual enzyme activity; class-IV are nearly normally active. Intriguingly, up to date, 85 class-I mutants, accounting for about 45% of the total, have been identified, but no null mutation has been reported. These observations suggest that G6PD protein could have other function(s) than the mediation of the robust oxPPP, which is indispensable to embryonic development. **b** Western blots to validate the knockout of G6PD in HeLa cells, and the re-expression of the empty vector (KO-EV), WT G6PD, or G6PD mutants (indicated as HeLa^KO^, HeLa^WT^, HeLa^R166H^, HeLa^R257G^, HeLa^242-243Δ^, and HeLa^H263A^). IDH1 and ME1 were also detected, and β-actin was used as the loading control. **c** Survival of HeLa cells with different states of G6PD, as indicated, after treatments either with 100 µM H_2_O_2_ for 8 h or 1 µM antimycin A for 24 h, or under hypoxia (0.5% O_2_) for 24 h, normalized to untreated cells. Here, one class-II variant, R166H, and two class-I mutants, 242-243Δ and R257G, were used. **d** The relative abundance of cellular 6-phospho-D-glucono-1,5-lactone (6PGL) by LC-MS/MS in HeLa cells with different states of G6PD, as indicated. **e** Mass isotopomer analysis of NADPH in HeLa cell lines, as indicated, cultured with the medium containing 10 mM of [3-^2^H] glucose for 8 h. **f** The NADPH/NADP^+^ ratio in HeLa cells with different states of G6PD, as indicated. **g** Cellular metabolites were measured by LC-MS/MS with an untargeted metabolomic method. Volcano plot of cellular metabolites in HeLa^KO^, relative to HeLa^WT^ cells. **h** Glucose uptake (left) and lactate excretion (right) in HeLa cells with different states of G6PD, as indicated, under the normal condition. Data were from triplicate experiments, and all experimental data were verified in at least two independent experiments. **i** NADH/NAD^+^ ratio in HeLa cells with different states of G6PD, as indicated, under the normal condition. **j** NADH/NAD^+^ ratio in HeLa^KO^ and HeLa^WT^ cells treated with antimycin A (1 µM) and/or α-KB (2 mM) for 4 h. **k** Survival of HeLa^KO^ cells treated with antimycin A (1 µM) or H_2_O_2_ (100 µM), without or with α-KB (1 or 5 mM), Pyruvate (1 or 5 mM), and OAA (1 or 5 mM) for 24 h, normalized to untreated cells. **l** Confirmation of Dox-induced expression of *Lb*NOX, mito*Lb*NOX, TPNOX, and mitoTPNOX in HeLa^KO^ cells. **m** Survival of cell lines from (G) treated with antimycin A (1 µM) in the presence or absence of Dox (50 nM, 24 h prior to antimycin A treatment) for 24 h, normalized to untreated cells. **n** Mass isotopomer analysis of isocitrate in HeLa^WT^, HeLa^KO^, and HeLa^R257G^ cells cultured with ^13^C_5_-glutamine for 4 h in the presence of antimycin A (1 μM). **o** Western blot analysis of G6PD, pACC1, ACC1, pAMPKα, and AMPKα in HeLa cells with different states of G6PD, as indicated, treated with 1 µM antimycin A for the time as indicated. GAPDH was used as the loading control. **p** Survival of HeLa^WT^ and HeLa^KO^ cells treated without or with 1 µM antimycin A for 24 h, in the presence or absence of 500 µM AICAR (pretreatment for 12 h) and/or 10 µM compound C. **q** Survival of HeLa/Cas9 and HeLa^IDH-DKO^ cells treated with 10 µM compound C and/or 1 µM antimycin A for 24 h. **r** The brief working model for the anti-stress roles of G6PD. Data were from triplicate experiments, and all experimental data were verified in at least two independent experiments. Error bars represent mean ± SD. *p < 0.05; **p < 0.01 (Student’s *t*-test).

We deleted G6PD in HeLa, MDA-MB-231, and HCT116 cells, and then re-expressed the wild type (WT) enzyme, some natural variants, and mechanism-based inactivated mutants^1^ (Fig. 1b and Supplementary Fig. S1a-c). G6PD-KO significantly sensitized cells to hydrogen peroxide (H_2_O_2_), hypoxia, and the electron transport chain (ETC) inhibition by antimycin A, which, however, was completely rescued by all the expressions except artificial mutants including H263A (Fig. 1c and Supplementary Fig. S1a-c), even though they displayed obviously decreased activities by measuring the product of 6-phosphate gluconolactone (Fig. 1d) and the [3-^2^H]-glucose-labeled NADPH m + 1 generated from oxPPP (Fig. 1e and Supplementary Fig. S1d). The NADPH/NADP^+^ ratio was dramatically reduced in HeLa^KO^ cells, but it was also totally reversed by natural mutants but not H263A (Fig. 1f). We meantime observed compensatively increased [3-^2^H]-glucose-labeled NADPH m + 2 in HeLa^KO^ and natural mutant-re-expressing cells (Fig. 1e), which resulted from the newly synthesized NADPH incorporating ribose 5-phosphated m + 2 derived from the non-oxPPP (Supplementary Fig. S1a). These data suggest that enabling the robust oxPPP is not required for the anti-stress activity of G6PD.

We then performed metabolomic profiling on HeLa^KO^, HeLa^WT^, and HeLa^R257G^ cells, and found that, except the products of the oxPPP, most of the significantly changed metabolites in HeLa^KO^ cells were reversed by the re-expression of R257G without detectable activity (Supplementary Fig. S2a-c). Among the significantly increased metabolites in KO cells, pyruvate and α-hydroxyglutarate were associated with glycolysis and center carbon metabolism (Fig. 1g), and the KEGG analysis also enriched the genes involved in these pathways (Supplementary Fig. S2d). Indeed, G6PD-KO promoted glucose uptake and lactate secretion, which were suppressed by re-expressing WT and R257G-G6PD, but not H263A mutant (Fig. 1h). The ^13^C_6_-glucose tracing revealed similar labeled fractions of all the metabolites tested in HeLa^WT^ and HeLa^KO^ cells, but increased cellular levels of glycerol 3-phosphate, lactate, acetyl-CoA, and α-hydroxyglutarate, and decreased levels of aspartate and all the intermediates in the tricarboxylic acid cycle except α-ketoglutarate in HeLa^KO^ cells (Supplementary Fig. S3a,b). These metabolic changes were exactly similar to those induced by NADH accumulation under ETC dysfunction.^2^ We further measured an increased NADH/NAD^+^ ratio in HeLa^KO^ but in HeLa^WT^ and HeLa^R257G^ cells (Fig. 1i), suggesting that G6PD can maintain the NADH/NAD^+^ homeostasis, independently of its intact activity.

Hypoxia, H_2_O_2_, and antimycin A treatments increased the NADH/NAD^+^ ratio (Supplementary Fig. S4a). To determine whether the deregulated NADH/NAD^+^ homeostasis contributed to cell death in G6PD-KO cells, we used α-ketobutyrate, a pyruvate analogue substantially converting NADH to NAD^+^,^3^ to reduce the NADH/NAD^+^ ratio (Fig. 1j and Supplementary S4b). We found that α-ketobutyrate, as well as other electron accepters, significantly protected G6PD-KO cells against antimycin A and H_2_O_2_ (Fig. 1k and Supplementary Fig. S4c).

Furthermore, we expressed a doxycycline-inducible *Lb*NOX^4^ (Fig. 1l), which transfers electrons from NADH to oxygen, in HeLa^KO^ cells. Both cytosolic *Lb*NOX and mitochondrial mito*Lb*NOX significantly decreased NADH/NAD^+^ ratio (Supplementary Fig. S4d, e) and repressed antimycin A-induced cell death (Fig. 1m). Another enzyme, TPNOX, can convert NADPH to NADP^+^ and thus decrease the NADPH/NADP^+^ ratio, but it also meantime reduced the NADH/NAD^+^ ratio.^5^ Interestingly, only the cytosolic TPNOX, not mitochondrial mitoTPNOX, rescued HeLa^KO^ cells (Fig. 1m). Therefore, G6PD-KO sensitizes cells to the deregulated NADH/NAD^+^ ratio, most likely in the cytosol, not to the decreased NADPH/NADP^+^.

Although lipid biosynthesis was thought to require a large amount of NADPH mainly afforded by the oxPPP, we found no difference in lipids and ^13^C_6_-glucose-labeled fatty acids between HeLa^KO^ and HeLa^WT^ cells (Supplementary Fig. S5a, b). However, our tracing results of ^13^C_5_-glutamine indicated that the metabolic flux of glutamine to fatty acids actually decreased in HeLa^KO^ cells (Supplementary Fig. S5c), although its contribution was much less than glucose in the normal condition. Glutamine carbon was usually used to synthesize fatty acid through the reductive pathway (Supplementary Fig. S5d), which was specifically promoted by ETC dysfunction.^2^ Our metabolic flux analyses of ^13^C_5_-glutamine in HeLa^WT^, HeLa^KO^, and HeLa^R257G^ cells showed that antimycin A obviously boosted reductive glutamine metabolism in HeLa^WT^ and HeLa^R257G^ but not HeLa^KO^ cells, indicated by the increased ^13^C_5_-glutamine-labeled fractions of isocitrate m + 5 (Fig. 1n).

Although the fraction and content of ^13^C_5_-glutamine-derived acetyl-CoA m + 2 were not promoted in HeLa^KO^ cells by antimycin A, the cellular level of acetyl-CoA did not decrease (Supplementary Fig. S5e,f), suggesting the presence of a compensative pathway, most likely from glucose metabolism. As expected, upon the antimycin A treatment, we observed an increase in ^13^C_6_-glucose-derived malate m + 3, citrate m + 3 and m + 5, and acetyl-CoA m + 2 in HeLa^KO^ cells (Supplementary Fig. S6a-d), demonstrating that HeLa^KO^ cells still utilize glucose, instead of glutamine, to synthesize acetyl-CoA even under ETC inhibition.

The biosynthetic process of acetyl-CoA from glucose produces NADH, whereas that from glutamine via the reductive pathway did not.^2^ This could underlie the deregulated NADH/NAD^+^ ratio in G6PD-KO cells.

We next deleted IDH1, as well as its mitochondrial isoform IDH2, to block reductive glutamine metabolism in HeLa cells, and observed the increased NADH/NAD^+^ ratio (Supplementary Fig. S7a). However, HeLa^IDH1/2-DKO^ cells did not sensitize to antimycin A (Fig. S7a). It suggests that blocking reductive glutamine metabolism is insufficient to explain the susceptibility of G6PD-KO cells to the stresses.

We then observed that antimycin A quickly induced the phosphorylations of AMPK and its substrate ACC1 in HeLa^WT^ and HeLa^R257G^, but not in HeLa^KO^ and HeLa^H263A^ cells (Fig. 1o and Supplementary Fig. S7b), suggesting that G6PD, independently of its intact activity, can effectively support AMPK activation. Furthermore, 5-amino-4-imidazolecarboxamide ribonucleoside (AICAR) was used to activate AMPK in HeLa, MDA-MB-231, and HCT116 cells, and confirmed that the phosphorylations of AMPK and ACC1 in G6PD-KO cells were much slower than those in G6PD-WT cells (Supplementary Fig. S7c). As expected, AICAR pre-treatment obviously protected G6PD-KO cells against death induced by antimycin A or H_2_O_2_ (Fig. 1p and Supplementary Fig. S7d), which was completely blocked by AMPK inhibitor compound C (Fig. 1p and Supplementary Fig. S7e). These data demonstrate that AMPK activation is also necessary for cells to cope with the stresses.

At last, we found that the increased NADH/NAD^+^ ratio could delay AMPK activation (Supplementary Fig. S8a-c), and verified that simultaneously blocking AMPK and reductive glutamine metabolism mimicked G6PD knockout in HeLa cells under the stress condition (Fig. 1q). Since the oxPPP products were not significantly detected in cells expressing R257G-G6PD, traces of metabolites derived from the leaking activity, or an alternatively unidentified function, of naturally mutated G6PD could function as signaling molecules to trigger cascades to activate AMPK and reductive glutamine metabolism, which is required to neutralize the stresses (Fig. 1r and Supplementary Fig. S8d). Taken together, our findings help us to better understand the physiological roles of G6PD and its association with human diseases.

## Supplementary information

Supplementary Files

## Data Availability

The data that support the findings of this study are available within the Article and its Supplementary Information or from the corresponding author upon reasonable request.
