# Supplementary material for: Glucose-6-phosphate dehydrogenase neutralizes stresses by supporting reductive glutamine metabolism and AMPK activation
Source: Signal Transduct Target Ther. 2021 Feb 4;6:46. doi: 10.1038/s41392-020-00399-x (PMC7859178; doi:10.1038/s41392-020-00399-x)
Supplement: Supplementary file 1 — Supplementary Files [file 41392_2020_399_MOESM1_ESM.docx]

**Supplementary Materials for**

**Glucose-6-phosphate dehydrogenase neutralizes stresses by supporting reductive glutamine metabolism and AMPK activation**

Benfu Zhong^1,2 #^, Dewei Jiang^3 #^, Yang Hong^1^, Lifang Li^1^, Li Qiu^1^, Ronghui Yang^2^, Xiaohan Jin^2^, Yawen Song^1^, Ceshi Chen^3^ *, Binghui Li^1,2^ *

1 Department of Cancer Cell Biology and National Clinical Research Center for Cancer, Tianjin Medical University Cancer Institute and Hospital, Tianjin 300060, P. R. China.

2 Department of Biochemistry and Molecular Biology, Capital Medical University, Beijing 100069, P.R. China.

3 Key Laboratory of Animal Models and Human Disease Mechanisms of Chinese Academy of Sciences and Yunnan Province, Kunming Institute of Zoology, Chinese Academy of Sciences, Kunming 650223, China.

^#^These authors contributed equally.

*Correspondence to: Binghui Li ([bli@ccmu.edu.cn](mailto:bli@ccmu.edu.cn)) or Ceshi Chen ([chenc@mail.kiz.ac.cn](mailto:chenc@mail.kiz.ac.cn))

**This PDF file includes:**

Materials and Methods

Figures. S1 to S8

Materials and Methods

**General reagents.** AICAR, compound C, ND-646 were obtained from MedChem Express (USA). ^13^C_6_-Glucose, [3-^2^H]-glucose, ^13^C_5_-glutamine and [2,4,5,6-^2^H]-nicotinamide were purchased from Cambridge Isotope Laboratories (USA). Antimycin A, phenformin, ZMP, rotenone, doxycycline, pyruvate, oxaloacetate, α-ketoglutarate, α-ketobutyrate, and all general chemicals were obtained from Sigma (USA) unless otherwise described.

**Cell culture.** HeLa, MDA-MB-231, HCT116 and HEK-293T cells were obtained from ATCC. All the cells were cultured in high glucose DMEM supplemented with 10% fetal bovine serum (BioInd, Israel) and 50 IU penicillin/streptomycin (Invitrogen, USA) in a humidified atmosphere with 5% CO_2_ at 37 °C. Hypoxia studies were carried out at 0.5% oxygen.

**Proliferation assay.** Cells were plated in triplicate in 12-well plates at 5×10^^^4 cells per well in 2 mL. After the desired treatments as indicated in experiments, wells were washed twice with PBS buffer to remove dead cells, and then the entire contents of the well were trypsinized. Cell number was determined using a hemocytometer.

**Stimulation of H_2_O_2_, antimycin A, phenformin, rotenone and hypoxia.** In all experimental researches in vitro, cells were incubated in defined-medium based on DMEM, which contains 10% FBS-Dialyzed, 50 IU penicillin/streptomycin, 25 mM glucose and 1 mM glutamine. Cells were triplicately plated onto 12-well dishes, with initial seeding density of 1.5×10^^^5 cells per well for HeLa, MDA-MB-231 and 3.0×10^^^5 cells for HCT116. After overnight or 24h incubation for cells to adhere, three wells were counted as the initial number at the time of treatment. Then cells were washed twice in phosphate buffered saline (PBS) and 2 mL of treatment media containing was added. Cells were treated with 100 µM H_2_O_2_ for 8h and with 1 µM antimycin A for 24h. As for the stimulation with phenformin and rotenone, the initial seeding density of cells in a well was 1.0×10^^^5 for HeLa, MDA-MB-231 and 2.0×10^^^5 for HCT116, 3 mL of treatment media containing 5 µM phenformin or 1 µM rotenone was added to each well, and cells were cultured for 48h. Hypoxia studies were carried out at 0.5% oxygen for 24h.

**Stimulation of AICAR, ZMP and compound C.** Cells were triplicately plated onto 12-well dishes, with initial seeding density of 2×10^^^5 cells per well for HeLa, MDA-MB-231 and 4.0×10^^^5 cells for HCT116. After overnight incubation for cells to adhere, cells were treated with 500 µM AICAR, 500 µM ZMP and/or 10 µM compound C for the desired period as described in the experiments. Notably, to rescue the G6PD knockout cells under the stimulations with antimycin A and H_2_O_2_, cells were pretreated with 500 µM AICAR for 12h and 500 µM ZMP for 24h.

**Plasmids and lentivirus production.** Expression plasmids for wild type and mutated G6PDs, SOD1-myc, SOD2-myc, Catalase-myc and Gpx1 were constructed in the pCDH-Neo-CMV or pCDH-puro-CMV vectors (Clontech, USA) for stably expression in cells. *Lb*NOX，mito *Lb*NOX, TPNOX and mitoTPNOX were constructed in pLVX-TRE3G-IRES (Clontech, USA), and then respectively expressed in cells expressed Tet3G for doxycycline-inducible expression. All plasmids were verified by sequencing (Genewiz, China). Viral packaging was done according to a previously described protocol([*42*](file:///E:\Cancer%20Biology\G6PD\G6PD%20paper\STTT0920\10-22修改\STTT-Supplymentary%20Figures-methods-%201022-Revision%20(make%20up).docx#_ENREF_42)). Briefly, expression plasmids, pCMV-dR8.91, and pCMV-VSV-G were co-transfected into 293T cells at a final concentration of 10 μM polyethylenimine, then media containing virus was collected 48 h after transfection and stored at -80 °C. Cancer cells were infected with the viruses at the titer of 100% infection in the presence of polybrene (10 μg mL^−1^) for 12-24 h, and then cells were selected with puromycin or neomycin.

**CRISPR knockout of G6PD and IDH1/2.** G6PD were deleted from HeLa, MDA-MB-231 and HCT116 cells, and both of IDH1 and IDH2 were knocked out in HeLa cells using the CRISPR–Cas9 system. pCDH-Cas9-2A-GFP-BSD was used to express Cas9. sgRNAs were cloned into pLentiGuide-puro-Vector([*14*](file:///E:\Cancer%20Biology\G6PD\G6PD%20paper\STTT0920\10-22修改\STTT-Supplymentary%20Figures-methods-%201022-Revision%20(make%20up).docx#_ENREF_14)) linearized with BsmBI. The target sequences for G6PD: GATCCGCGTGCAGCCCAACG; for IDH1: GCATGACGACCTATGATGAT; for IDH2: ATGAGATGACCCGTATTATC. Cells were co-transfected with the plasmids expressing sgRNA and Cas9-EGFP, and then were cultured for 48h. Single cells were seeded in a well of 96 wells plate by FACS based on green fluorescence and allowed to grow until colonies formed. Colonies were picked and expanded until large enough to allow freezing of stocks and analysis of G6PD, IDH1 and IDH2 expression by western blot and/or PCR fragments sequencing.

**NADH/NAD^+^ and NADPH/NADP^+^ ratio assay.** NADH/NAD^+^ and NADPH/NADP^+^ were measured using the NAD/NADH-Glo^TM^ Assay and NADP/NADPH-Glo^TM^ Assay (Cat#G9072/G9082 from Promega), according to the manual instructions with modifications. Briefly, 1.5×10^5 cells per well were seeded in 12-well plates for overnight or 24h, and then incubated with the treatment medium for the desired period. Cells were quickly washed once with PBS, and then extracted with 400 uL ice-cold lysis buffer. To measure NADH or NADPH, 150 μL of the samples was moved to wells of a 48-well plate and incubated at 60 °C for 15 min. To measure NAD^+^ or NADP^+^, another 150 μL of the samples was moved to wells of another 48-well plate containing 75 μL of 0.4 N HCl and incubated at 60 °C for 15 min. Samples were equilibrated to room temperature and then each was quenched by neutralizing with 150 μL of HCl/Trizma solution (for NADH or NADPH measurement) or 75 μL of 0.5 M Tris-base (for NAD^+^ or NADP^+^ measurement). 20 μL of the neutralized samples was moved to wells of a 384-well solid black luminometer plate (Cat#3570 from Corning), and mixed with 20 μL of the newly prepared NAD/NADH-Glo^TM^ or NADP/NADPH-Glo^TM^ Detection Reagent. The mixtures were gently shaken for 50 minutes at room temperature, and then the luminescence was measured using a Synergy H1 HybridMulti-Mode reader (BioTek, USA). To acquire the relative abundance of NAD^+^, NADH, NADP^+^ and NADPH in cells lysis, the measured signals were normalized by cell number from the parallel experiments.

**Glucose uptake and lactate excretion in cells.** Measurement of lactate and glucose concentrations in cell culture medium was performed using the M-100 Automatic Biosensors Analyzer (Shenzhen SiemenTechnology Co., Ltd) according to user instructions. Cell preparations and detection procedures were described as before([*12*](file:///E:\Cancer%20Biology\G6PD\G6PD%20paper\STTT0920\10-22修改\STTT-Supplymentary%20Figures-methods-%201022-Revision%20(make%20up).docx#_ENREF_12)). Briefly, cells were grown in 12-well plates at 2×10^5^ cells per well, and then were incubated with the treatment medium containing 10% dialyzed FBS, 10 mM glucose, 1 mM glutamine and no pyruvate for 8h. Cell media were collected at 0 h and 8h. Aliquots of 100 µL medium were used to measure the concentration of glucose and lactate. The increased or reduced amount of metabolite in the medium, normalized for area under the curve, was the excretion or uptake of metabolite by cells per hour. The values were either directly presented or normalized for comparison with the control.

**Immunoblotting.** Western detection was performed using a Li-Cor Odyssey image reader or a C-DiGit Chemiluminescence Western Blot Scanner. The goat anti-rabbit IgG (Cat#P/N926–68071, 1:10 000 dilution) and goat anti-mouse IgG (Cat#P/N926–32210, 1:10 000 dilution) secondary antibodies were obtained from Li-Cor. The goat anti-rabbit IgG/HRP (Cat #ZDR-5306, 1:5,000 dilution) and the goat anti-mouse IgG/HRP (Cat#ZDR-5307, 1:5 000 dilution) secondary antibodies were obtained from ZSGB-Bio. GAPDH (Cat#60004-1-lg, 1:5 000 dilution), NRF2 (Cat#16396-1-AP, 1:1000 dilution), IDH1 (Cat#12332-1-AP, 1:1 000 dilution), IDH2 (Cat#15932-1-AP, 1:1000 dilution), ME1 (Cat#16619-1-AP, 1:1 000 dilution), ACC1 (Cat#21923-1-AP, 1:5000 dilution) and c-MYC (Cat#10828-1-AP, 1:5 000 dilution) antibodies were obtained from Proteintech. FLAG (Cat#MBL-3L, 1:10 000 dilution) was obtained from MBL Bio.GPX1 (Cat#DF6249, 1:500 dilution) was obtained from Affinity Bio. pAMPKα (Thr172, Cat# 2535, 1:1000 dilution ), pACC1 (Ser79, Cat# 3661, 1:1000 dilution ) and AMPKα (Cat# 2532, 1:1000 dilution ) were obtained from CST.

**RNA-seq and data analysis.** Construction of an RNA-seq library and RNA-seq was completed by Beijing Novogene Biological Information Technology Co., Ltd (Beijing, China). The sequencing library was determined by NEBNext® Ultra RNA Library Prep Kit for Illumina®, and sequencing was performed on an Illumina Novaseq 6000. Raw reads of fastq format were firstly processed through in-house perl scripts. The quality control of samples was accomplished using FASTQC. Clean reads were aligned to the human reference genome HG19 by Hisat2 v2.0.5. FeatureCounts v1.5.0-p3 was used to calculate gene expression, and DESeq2 was used to determine differential expression. Differential expressed genes with log2 (fold change) >1 and false discovery rate <0.001 were considered significant, and then KEGG enrichment analysis was performed.

**Metabolites isotope tracing.** LC-MS/MS analyses were conducted on a TSQ Quantiva triple quadrupole mass spectrometer networked to a Dionex UltiMate 3000 UPLC system (Thermo Fisher Scientific) at the Metabolomics Facility at Tsinghua University Branch of China National Center for Protein Sciences (Beijing, China). Multiple reaction monitoring mode was developed using chemical standards. Experiments were performed in medium containing 10% dialyzed FBS. DMEM lacking glucose, glutamine, pyruvate and phenol red was prepared from powder (Cat#DMP52 from Casson Labs) by adding 3.7 g NaHCO3 per liter and adjusting the pH to 7.4, and then supplemented with 10 mM ^13^C_6_-glucose and 1 mM unlabeled glutamine or 10 mM unlabeled glucose and 1 mM ^13^C_5_-glutamine or 10 mM [3-^2^H]-glucose and 1 mM unlabeled glutamine. For nicotinamide labeling, 32.8 μM [2,4,5,6-^2^H]-nicotinamide was added to the phenol red free RIPM1640 (BioInd, Israel), which intrinsically contains 11.1 mM glucose, 2.05 mM glutamine and 8.2 μM label-free nicotinamide. As for polar metabolite analysis, cells were grown in 60-mm dishes (100-mm dishes for NADPH isotope tracing) until 80% confluent, then rinsed with PBS and cultured with 2 mL ^13^C-containing medium for 8 h under normal condition or in the presence of 1 µM antimycin A (6.5 mL ^2^H-containing medium for 8 h under normal condition for NADPH isotope tracing). Cells were extracted by freeze-thawing three times in 0.5 mL 80% methanol (prechilled to -80°C and 1 mL for 100-mm dishes) after they were rinsed twice with room temperature PBS. Macromolecules and debris were removed by centrifugation at 14,000 g for 20 min at 4°C, and the metabolite-containing supernatants were dried under nitrogen gas. Dried samples were stored at -80°C and then resuspended in 50 µL water and prepared for LC-MS/MS analyses. 1 µL of each sample was injected onto a Synergi Hydro-RP 100A 2.1×100-mm column (Phenomenex) for metabolite separation with column temperature at 35°C. Mobile phases A and B were 10 mM thiobarbituric acid in aqueous with pH 5.0 and 100% methanol, respectively. The chromatographic gradient was set for mobile phase B as follows: 0-3.5 min: 1% B; 3.5-22 min: from 1% to 70% B; 22-23 min: from 70% to 90% B; 23-25 min: 90% B; 25-30 min: 1% B. Data were acquired using a positive/negative switching method. Spray voltages of 3.5 kV and 2.5 kV were applied for positive and negative modes, respectively. Q1 and Q3 resolution was set at 0.7, and 1 s of cycle time was used in the method. The abundance of each mass isotopomer was then mathematically corrected to eliminate natural abundance isotopes and finally converted into a percentage of the total pool.

**Untargeted metabolomics.** Untargeted metabolites screening is performed on Q Exactive Orbitrap mass spectrometer (Thermo Fisher Scientific) with positive/negative ion switching at the Metabolomics Facility at Tsinghua University Branch of China National Center for Protein Sciences(Beijing, China). HeLa^KO^, HeLa^WT^ and HeLa^R257G^ Cells were grown in 100-mm dishes until 80% confluent, then rinsed with PBS and cultured with 8 mL phenol red free DMEM that containing 10% dialyzed FBS, 25 mM glucose and 1 mM glutamine for 24 h. Then samples were prepared as described in the section of “Metabolites isotope tracing”, and samples were randomly detected, in order to avoid machine drift. In positive mode, BEH amide column (Waters, USA) is used for separation with column temperature at 35°C. Mobile phase A is prepared by dissolving 0.63g of ammonium formate in 50mL of HPLC-grade water, which is then mixed with 950mL of HPLC-grade acetonitrile and 1μL of formic acid. Mobile phase B is prepared by dissolving 0.63g of ammonium formate in 500mL of HPLC-grade water, followed by the addition of 500 mL of HPLC-grade acetonitrile and 1μL formic acid. Separation is initiated at 1% mobile phase B with flow rate at 300μl/min. The chromatographic gradient was set as follows: 0-3.5 min: 99% A and 1% B; 3.5-17 min: 80% A and 20% B; 17-17.5 min: 20% A and 80% B; 17.5-19 min: 1% A and 99% B; 19.1-20 min: 99% A and 1% B. In negative mode, BEH C18 column (Waters, USA) is used. Column temperature is 35°C. Mobile phase A is prepared by dissolving 0.3953g of ammonium bicarbonate in 1 L of HPLC-grade water (pH~8.0). Mobile phase B is HPLC-grade acetonitrile. Gradient starts at 1% mobile phase B with flow rate at 250μl/min，and shown as follows：0-10 min: 99% A and 1% B; 10-17 min: 1% A and 99% B; 17.1-20 min: 99% A and 1% B. The detailed mass spectrometer parameters are shown as follows: spray voltage (kV), 3.5 ESI+ for positive and 3.0 ESI- for negative; capillary temperature, 320°C for both; sheath gas flow rate, 35 Arb for both; aux gas flow rate, 8 Arb for positive and 12 Arb for negative; mass range (m/z), 70-1050 for positive and 80-1200 for negative; full ms resolution, 70000 for positive and 60000 for negative; MS/MS resolution, 17500 for positive and 30000 for negative; TopN, both of 10; NCE/stepped NCE, 15,30 and 45 for both; Duty cycle, both of 1.2 s. MRM data were analyzed using Tracefinder 3.2 (Thermo Fisher Scientific) to quantify metabolites. In-house library containing MS/MS spectra of over 1500 metabolites were used to assign metabolite ID. Two levels of identification were achieved in this method, one with MS/MS confirmation based on library fragment match; the other assigned solely according to the accurate mass of precursor ions (< 10 ppm mass tolerance). The “library score” was applied to show the confidence of MS/MS confirmation. Only the compounds with “library score” > 30 were considered as confident identification. For the multivariate statistical analysis, the MetaboAnalyst (<http://www.metaboanalyst.ca>) web-based system was used.

**Isotope tracing of fatty acids.** Fatty acids analysis was performed on Q Exactive orbitrap mass spectrometer (Thermo, CA) at the Metabolomics Facility at Tsinghua University Branch of China National Center for Protein Sciences(Beijing, China). HeLa cells were grown in 60-mm dishes with the initial seeding density of 6×10^^^5 cells, and then were incubated normally for 48 h in the defined-medium based on DMEM, which contains 10% FBS-Dialyzed, 50 IU penicillin/streptomycin, 20mM [U-^13^C]-glucose and 2mM glutamine or 20mM glucose and 2mM [U-^13^C]-glutamine. Samples were prepared as previously described([*43*](file:///E:\Cancer%20Biology\G6PD\G6PD%20paper\STTT0920\10-22修改\STTT-Supplymentary%20Figures-methods-%201022-Revision%20(make%20up).docx#_ENREF_43)). Media were aspirated, and cells were rinsed twice with 2 mL room temperature PBS and then extracted with 1 mL 50% methanol solution containing 0.1 M HCl (prechilled to -80°C). The resulting liquid and cell debris were scraped into a glass vial. Chloroform (0.5 mL) was added, the mixture was vortexed for 1 min and then centrifuged at 3,000 rpm for 15 min, and the chloroform layer was transferred to a glass vial. The extract was dried under N_2_, reconstituted into 1 mL 90% methanol solution containing 0.3 M KOH, incubated at 80°C for 1 h to saponify fatty acids, acidified with 0.1 mL of formic acid, extracted twice with 1 mL of hexane, and dried under N_2_. Dried samples were stored at -80°C and then resuspended in 150 µl dichloromethane:methanol and prepared for LC/MS analyses([*44*](file:///E:\Cancer%20Biology\G6PD\G6PD%20paper\STTT0920\10-22修改\STTT-Supplymentary%20Figures-methods-%201022-Revision%20(make%20up).docx#_ENREF_44)). Cortecs C18 column (2.1×100 mm; Waters) was applied for analysis. Mobile phase A was prepared by dissolving 0.77 g of ammonium acetate in 400 mL of HPLC-grade water, followed by adding 600 mL of HPLC-grade acetonitrile. Mobile phase B was prepared by mixing 100 mL of acetonitrile with 900 mL isopropanol. The gradient was as below: 0 min, 37% B; 1.5 min, 37% B; 4 min, 45% B; 5 min, 52% B; 8 min, 58% B; 11 min, 66% B; 14 min, 70% B; 18 min, 75% B; 20 min, 98% B; 22 min, 98% B; 22.1 min, 37% B; 25 min, 37% B. Some MS parameters were different from polar compound analysis as below: spray voltage, 3.2 kV for positive and 2.8 kV for negative; capillary temperature, 320°C; aux gas flow rate (arb), 10; and mass range (m/z), 240-2,000 for positive and 200-2,000 for negative. Multiple reaction monitoring data were analyzed using Tracefinder. Lipids were assigned by home-built lipid database in “screening” mode and quantified in “quan” mode. Lipids were identified based on matching precursor and characteristic fragment masses. 5-ppm and 10-ppm mass tolerance was used for precursor and fragment, respectively. Only the lipids with chromatographic area >5E6 were considered as confident identification. A 0.25-min retention time shift was allowed for quantitation. Retention times and mass fragmentation signatures of all metabolites were validated using pure standards. Ion pairs with various ^13^C labels were derived based on chemical structures of precursors and fragments. The abundance of each mass isotopomer was then mathematically corrected to eliminate natural abundance isotopes and finally converted into a percentage of the total pool. The percentage of labeled fatty acid carbons of the total pool (P) was calculated as the following([*13*](file:///E:\Cancer%20Biology\G6PD\G6PD%20paper\STTT0920\10-22修改\STTT-Supplymentary%20Figures-methods-%201022-Revision%20(make%20up).docx#_ENREF_13)):

$$P=\sum_{n=1,2,3\ldots m}^{m} \frac{n}{m}*P_{n}$$

where n is the number of labeled carbons in an m-carbon fatty acid or cholesterol and P_n_ is the percentage of n-carbon-labeled fatty acid of the total pool. The areas of the ion peaks of interest were corrected by cell number. Finally, the relative abundance of metabolites was compared with each other.

**Lipids targeted metabolomics.** Lipids analysis was performed on Q Exactive orbitrap mass spectrometer (Thermo, CA) at the Metabolomics Facility at Tsinghua University Branch of China National Center for Protein Sciences (Beijing, China). Briefly, HeLa cells were grown in 60-mm dishes with the initial seeding density of 6×10^^^5 cells, and then were incubated normally for 48h in the defined-medium based on DMEM, which contains 10% FBS-Dialyzed, 50 IU penicillin/streptomycin, 20mM glucose and 2mM glutamine. Media were aspirated, and cells were rinsed twice with 2 mL room temperature PBS and then scraped with 1 mL room temperature PBS. The resulting liquid and cell debris were transfered into a glass vial. 4 mL CHCl_3_:MeOH (2:1) was added, the mixture was vortexed for 1 min three times and then centrifuged at 3,000 rpm for 15 min. The chloroform layer was transferred to a glass vial. The extract was dried under N_2_. Dried samples were stored at -80°C and detected as described in the section of “Isotope tracing of fatty acids flux”. Lipids were identified and quantified using LipidSearch 4.1.30 (Thermo, CA). Mass tolerance of 5 ppm and 10 ppm were applied for precursor and product ions. Retention time shift of 0.25 min was performed in “alignment”. M-score and chromatographic areas were used to reduce false positives.

**Statistical analysis.** Data are given as means ± SD. Statistical analyses were performed using unpaired two-tailed Student’s t test for comparison between two groups. Asterisks in the figures indicated statistical significances (*, P < 0.05; **, P < 0.01).

Figure. S1.

**Figure. S1 The robust oxPPP activity of G6PD is dispensable to its antioxidant ability. a** Survival of HeLa cells with different state of G6PD, as indicated, treated with 100 µM H_2_O_2_ for 8 h or 1µM antimycin A for 24 h, normalized to untreated cells. **b-c** Cell survival of MDA-MB-231 (c) and HCT116 (d) cells with different states of G6PD, as indicated, after treatments with 100 µM H_2_O_2_ for 8 h or 1µM antimycin A for 24 h, normalized to untreated cells. **d** Schematic for the metabolic flux of [3-^2^H]-glucose in the oxidative PPP and the non-oxidative PPP. Data were from triplicate experiments, and all experimental data were verified in at least two independent experiments. Error bars represent mean ± SD. **, *p* < 0.01 (Student’s *t*-test).

Figure. S2.

**Figure. S2 The untargeted metabolomics analysis in HeLa cells. a** The principal component analysis (PCA) of raw data obtained from the untargeted metabolomics experiment in HeLa^KO^, HeLa^WT^ and HeLa^R257G^ cells. These results showed that the samples of HeLa^KO^ were sharply separated from those of HeLa^WT^ and HeLa^R257G^, while HeLa^R257G^ was close to but different from HeLa^WT^, indicating that R257G mutant can restore most of the functions of WT G6PD. This speculation was confirmed by further analysis that except the products of the oxPPP, most of significantly changed metabolites in HeLa^KO^ cells were reversed by the re-expression of R257G (**b,c**). **b** Cellular metabolites were measured by LC-MS/MS with an untargeted metabolomic method. **c** Representative metabolites significantly changed across HeLa^KO^, HeLa^WT^ and HeLa^R257G^ cells. **d** KEGG pathway enrichment in HeLa^KO^, relative to HeLa^WT^ cells, based on RNA-Sequencing data. Data were from three independent cultures. Data were from triplicate experiments, and all experimental data were verified in at least two independent experiments.

Figure. S3.

**Figure. S3** **The glucose-labeled metabolic flux in HeLa cells. a** Mass isotopomer analysis of 6-phospho-D-glucono-1,5-lactone (6PGL), phosphoribosyl pyrophosphate (PRPP), Glycerol-3-phosphate, serine, lactate, acetyl-CoA, α-hydroxyglutarate (α-HG), and aspartate (Asp) in HeLa^KO^ and HeLa^WT^ cells cultured with the medium containing 10 mM of ^13^C_6_-glucose for 8 h. **b** Mass isotopomer analysis of ITP, ATP, citrate, aconitate, isocitrate, α-ketoglutarate, succinate, malate, and fumarate in HeLa^WT^ and HeLa^KO^ cells cultured with the medium containing 10 mM of ^13^C_6_-glucose for 8 h. We observed the similar labeled fractions of all the metabolites tested here. As expected, we did not detect the product of oxPPP, 6-phosphate gluconolactone (**a**), but the downstream products of ribose 5-phosphate (the most important product of PPP), ITP and ATP, kept unchanged (**b**). These data indicated that the non-oxPPP alternatively supplies the oxPPP-derived intermediates for biosynthesis. Data were from three independent cultures. Error bars represent mean ± SD. *, *p* < 0.05; **, *p* < 0.01 (Student’s *t*-test).

Figure. S4.

**Figure. S4 Stress-induced NADH/NAD^+^ ratio boost can be rescued by *Lb*NOX system. a** NADH/NAD^+^ ratio in HeLa cells under normoxia or hypoxia (0.5% O_2_) for 8h (*left panel*), or treated with 100 µM H_2_O_2_ for 1h or 1 µM antimycin A for 4h (*right panel*). **b** NADH/NAD^+^ ratio and NADPH/NADP^+^ ratio in MDA-MB-231 or HCT116 cells, with different states of G6PD as indicated, treated without or with 1 µM antimycin A for 4h. **c** Survival of MDA-MB-231^KO^ or HCT116^KO^ cells treated with 1 µM antimycin A for 4h or 100 µM H_2_O_2_ for 8h, in the presence or absence of 2 mM α-KB. **d-e** NADH/NAD^+^ and NADPH/NADP^+^ ratio of HeLa^KO^ cells expressing *Lb*NOX (d) or mito*Lb*NOX (e) treated with antimycin A (1 µM) in the presence or absence of Dox (50 nM, 24 h prior to antimycin A treatment) for 4h.Data were from triplicate experiments, and all experimental data were verified in at least two independent experiments. Error bars represent mean ± SD. *, *p* < 0.05; **, *p* < 0.01 (Student’s *t*-test).

Figure. S5.

**Figure. S5 G6PD enables reductive glutamine metabolism. a** Lipidomics analysis of relative abundance of lipids, as indicated, in HeLa^WT^ and HeLa^KO^ cells. **b** Relative labeled carbons in fatty acids in HeLa^KO^ and HeLa^WT^ cells cultured with ^13^C_6_-glutamine for 48h in the normal condition. c Mass isotopomer analysis of labeled fatty acid carbons from ^13^C_6_-glucose in HeLa^WT^ and HeLa^KO^ cells cultured with medium containing 10 mM ^13^C_5_-glucose for 48h in the normal condition. **d** Schematic of glutamine oxidative or reductive metabolism. Black arrows show oxidative glutamine metabolism; red arrows show reductive glutamine metabolism. **e** Mass isotopomer analysis of malate, acetyl-CoA, citrate, isocitrate and α-ketoglutarate in HeLa^WT^, HeLa^KO^ and HeLa^R257G^ cells cultured with ^13^C_5_-glutamine for 4 h in the presence of antimycin A (1 μM). Antimycin A obviously boosted reductive glutamine metabolism in HeLa^WT^ and HeLa^R257G^ but not in HeLa^KO^ cells, indicated by the increased ^13^C_5_-glutamine-labeled fractions of isocitrate m+5, citrate m+5, malate m+3 and acetyl-CoA m+2. Our results also showed that conversion of α-ketoglutarate to isocitrate was substantively blocked in HeLa^KO^ cells treated with antimycin A. **f** The relative abundance of total and ^13^C-labeled acetyl-CoA in HeLa^WT^, HeLa^KO^ and HeLa^R257G^ in the condition as described in (e). Data were from three independent cultures. Error bars represent mean ± SD. *, *p* < 0.05; **, *p* < 0.01 (Student’s *t*-test).

Figure. S6.

**Figure. S6 G6PD loss does not reduce lipogenesis in the normal condition but suppresses the reductive glutamine metabolism. a-b** Schematic of glucose flux to acetyl-CoA in the conditions depending on the intermediates of the tricarboxylic acid (TCA) cycle (a) or independent of the TCA cycle (b). If ^13^C_6_-glucose can be oxidized through the TCA cycle, we would expect the labeled citrate m+2, m+3, m+4 and m+5, the director precursor of acetyl-CoA, and malate m+2 and m+3, the precursor of oxaloacetate (undetectable in cells) for citrate synthesis (a). Alternatively, ^13^C_6_-glucose-derived pyruvate is converted to malate m+3 or oxaloacetate m+3 for the synthesis of citrate m+5 and m+3 (b), when the TCA cycle is inactivated. **c-d** Mass isotopomer analysis of malate, acetyl-CoA, citrate, isocitrate, α-ketoglutarate and succinate in HeLaKO and HeLaWT cells cultured with 13C_6_-glucose for 4 h in the presence of antimycin A (1 μM). We used ^13^C_6_-glucose to trace the biosynthesis of acetyl-CoA in HeLa^WT^ and HeLa^KO^ cells treated with antimycin A. As expected, our results showed that upon the antimycin A treatment, almost no labeled iscictrate, α-ketoglutarate and succinate were detected, suggesting that glucose is not oxidized in the TCA cycle under such a condition. Data were from three independent cultures. Error bars represent mean ± SD. **, *p* < 0.01 (Student’s t-test).

Figure. S7.

**Figure. S7** **G6PD supports AMPK activation independently of its intact dehydrogenase activity. a** Western blots to validate the double knockout of IDH1 and IDH2 in HeLa cells; NADH/NAD^+^ ratio in HeLa/Cas9 and HeLa^DKO^ cells treated without or with antimycin A (1 µM) for 4h; Survival of HeLa/Cas9 and HeLa^DKO^ cells treated without or with antimycin A (1 µM) for 24h, normalized to untreated cells. Here, we observed that the NADH/NAD^+^ ratio in HeLa^IDH1/2-DKO^ cells, compared to the control cells, was significantly increased and further boosted by antmycin A, exactly similar to that in G6PD-KO cells. However, HeLa^IDH1/2-DKO^ cells did not sensitize to antimycin A, unlike the G6PD-knockout HeLa cells. **b** Western blot analysis of G6PD, pACC1, ACC1, pAMPKα and AMPKα in HeLa cells with different states of G6PD, as indicated, treated with 1 µM antimycin A for the time as indicated. GAPDH was used as the loading control. **c** Western blot analysis of G6PD, pACC1, ACC1, pAMPKα and AMPKα in HeLa^WT^ and HeLa^KO^ cells (*left pannel*), MDA-MB-231^WT^ and MDA-MB-231^KO^ cells (*middle pannel*)or HCT116^WT^ and HCT116^KO^ cells (*right pannel*), respectively, treated with 500 µM AICAR for the time as indicated. GAPDH was used as the loading control. **d** Survival of MDA-MB-231^KO^ cells and HCT116^KO^ cells treated without or with 1 µM antimycin A for 24h or 100 µM H_2_O_2_ for 8h, in the presence or absence of 500 µM AICAR (pretreatment for 12h). **e** Western blot analysis of G6PD, pACC1, ACC1, pAMPKα and AMPKα in HeLa^KO^ cells, treated without or with 1 µM antimycin A for 24 h, in the presence or absence of 500 µM AICAR (pretreatment for 12h) and/or 10 µM compound C (*Related to Fig. 1p*). Data were from triplicate experiments, and all experimental data were verified in at least two independent experiments. Error bars represent mean ± SD. *, *p* < 0.05; **, *p* < 0.01 (Student’s *t*-test).

Figure. S8.

**Figure. S8 The increased NADH/NAD+ ratio delays AMPK activation.** **a** NADH/NAD^+^ ratio in HeLa^WT^ and HeLa^KO^ cells treated with 1 µM antimycin A for 4h in the presence or absence of 500 µM AICAR (pretreatment for 12h). ACIAR can decrease the NADH/NAD^+^ ratio in HeLa^WT^ but not in HeLa^KO^ cells. **b** Western blot analysis of pACC1, ACC1, pAMPKα and AMPKα in HeLa^WT^ and HeLa^KO^ cells treated without or with 1 µM antimycin A for 8h in the presence or absence of 2 mM α-ketobutyrate. GAPDH was used as the loading control. α-Ketobutyrate, the attenuator of NADH/NAD^+^, apparently enhanced the levels of phosphorylated AMPK and ACC1 proteins in both HeLa^WT^ and HeLa^KO^ cells when they were treated with antitmycin A. These data in (**a,b**) suggest that the contribution of the deregulated NADH/NAD^+^ homeostasis to cell death of G6PD-knockout cells upon stresses could be mediated, at least in part, by its repression on AMPK activation. **c** Western blot analysis of pACC1, ACC1, pAMPKα and AMPKα in HeLa/cas9 and HeLa^IDH-DKO^ cells treated without or with 1 µM antimycin A (left) or 500 µM AICAR (right) for the time as indicated. GAPDH was used as the loading control. IDH1/2-double knockout indeed delayed the phosphorylations of AMPK and ACC1 in HeLa cells treated with antimycin A by about one hour (**c**, left). However, IDH1/2-double knockout did not affect the phosphorylated AMPK and ACC1 in HeLa cells induced by AICAR (**c**, right). These data suggest that disabling reductive glutamine metabolism does not influence AMPK activity if the NADH/NAD^+^ ratio is not enhanced. **d** The working model for the anti-stress roles of G6PD. In the presence of G6PD, when the stimuli, such as oxidative stress, hypoxia and ETC inhibition, act on cells, the resultant increased NADH/NAD^+^ ratio will drive the metabolic reprogramming, including reductive glutamine metabolism that can attenuate the accumulated NADH and glucose uptake that can be fermented to lactate to provide energy without NADH being generated. In the absence of G6PD, upon these stimuli, cells fail to initiate IDH1-dependent reductive glutamine and promote AMPK-mediated glucose uptake, and finally undergo death. The identified functions of G6PD do not rely on its intact dehydrogenase activity. Now, it seems that G6PD can support AMPK activation and reductive glutamine metabolism in the stress conditions, and moreover these functions do not require the intact dehydrogenase activity and the robust oxPPP. The red blood cells rely on the robust oxPPP, most likely because they lack the complicated metabolism that could not provide substitutive NADPH and not be rewired to adapt the stresses. In contrast, somatic cells can reprogram their metabolic pathways to neutralize the stresses, which requires G6PD, even with a very low activity, to support AMPK activation and reductive glutamine metabolism, complementary to its typical action mediating the robust oxPPP. Error bars represent mean ± SD. *, *p* < 0.05; **, *p* < 0.01 (Student’s *t*-test).
